# Living With Spasticity During the COVID‐19 Pandemic: A Qualitative Study of Patient, Carer and Physician Experiences

**DOI:** 10.1111/hex.70032

**Published:** 2024-09-23

**Authors:** Mohamed Sakel, Karen Saunders, Rafey Faruqui, Jamie Keene, David Wilkinson

**Affiliations:** ^1^ East Kent Hospitals University NHS Foundation Trust Canterbury UK; ^2^ Central England Rehabilitation Unit, South Warwickshire University NHS Foundation Trust Leamington Spa Hospital Warwickshire UK; ^3^ Centre for Health Services Studies, School of Social Policy, Sociology and Social Research, Division for the Study of Law, Society and Social Justice University of Kent Canterbury UK; ^4^ Department of Psychiatry Kent and Medway NHS and Social Care Partnership Trust Maidstone UK; ^5^ King Edward VI Five Ways School Birmingham UK; ^6^ School of Psychology University of Kent Canterbury UK

**Keywords:** botulinum toxin, COVID‐19 pandemic, lived experience, long‐term neurological condition, neuro‐rehabilitation, qualitative research, spasticity

## Abstract

**Background:**

Approximately 4.4 million people in England (8% of the total population) are living with a long‐term neurological condition. Within this group of vulnerable individuals, there will be individuals living with severe spasticity that requires regular outpatient treatment with botulinum toxin injection. The closure of outpatient spasticity services during the pandemic impacted individuals who required spasticity treatment and their carers, as well as the specialist clinicians responsible for service delivery.

**Objectives:**

We aimed to gain insight into the experiences of individuals living with spasticity, their carers and a clinical spasticity service lead during the pandemic, and to reflect on potential learning for the future.

**Methods:**

A qualitative study was designed using semi‐structured interviews conducted by telephone. Participants comprised patients living with a long‐term neurological condition who attended outpatient spasticity clinics before the start of the pandemic in England, primary carers who accompanied patients attending these clinics and a clinical spasticity service lead. Data were audio recorded, transcribed, anonymised and coded. Data analysis utilised the One Sheet of Paper thematic approach to identify themes, which were discussed and analysed by the interdisciplinary research team and two patient and carer participants.

**Results:**

Out of the 11 participants recruited, aged 36–77 years, seven comprised people living with spasticity related to a long‐term neurological condition, three were carers and one was a clinical spasticity service lead. Six participants were male and five were female. Among the participants, four were stroke survivors, two were living with spinal cord injury and one was living with multiple sclerosis. Analysis revealed six major themes: experience of living with spasticity during the pandemic; impact of the pandemic on patient, carer and clinician health; access to and experience of outpatient clinic appointments; coping strategies during the pandemic; system improvements; and learning from the pandemic period.

**Conclusion:**

These findings contribute research knowledge to a very limited research knowledge base and suggest that there is scope for improving system and service delivery through the allocation of research funding to senior clinicians working in this specialist area.

## 1 Introduction

The COVID‐19 pandemic has changed lives and left an indelible impact on many people, including those living with long‐term neurological conditions (LTNCS). From March 2020 to December 2021, the UK government imposed a series of lockdowns and other measures to gain control of the pandemic [1]. Although it is known that rehabilitation services were negatively impacted worldwide [2], there is very limited knowledge of the impact of the pandemic on people who were living with chronic neurological conditions at the time, which required regular outpatient (OP) treatment for effective symptom management. LTNC have been identified as a wide range of conditions caused either by injury or disease of the nervous system, which individuals will live with for the rest of their lives [3]. LTNC include those with sudden onset (e.g., stroke or traumatic brain injury), those which are progressive (e.g., multiple sclerosis [MS]) and those which are considered ‘stable’ [3, p. 9], although individuals may experience different needs over time due to growing older (e.g., adult cerebral palsy). In terms of prevalence, it is estimated that about 2.2 million [4] people live with progressive LTNC in England and about 2.2 million [4] people with an LTNC of sudden onset [4], which equates to 4.4 million people (8% of the total population of England) [4]. This is a large population group that has implications for health and social care services, and it is this group of individuals, who are most likely to experience difficulties related to spasticity.

Spasticity is a common clinical symptom experienced by people living with LTNC such as traumatic brain injury, stroke, spinal cord injury and MS, which creates difficulties and can interfere with physical, psychological and social abilities [5]. Spasticity is a clinical phenomenon or symptom caused by injury or damage to the central nervous system (brain and spinal cord), which is frequently described as a feature of the upper motor neuron (UMN) syndrome [6]. Spasticity has a number of definitions in the literature (Table 1) [7, 8, 9, 10]. In terms of impact, an international online survey of 121 people living with spasticity undertaken pre‐pandemic found that 72% of respondents reported that spasticity had an adverse impact on their quality of life, 44% reported that it had an adverse impact on their level of independence and 44% reported they experienced depression [11, p. 1428]. Additionally, 64% of respondents had family members who were providing care support, and 73% received treatment with botulinum toxin (BT) to manage their spasticity [11, p. 1428].

**Table 1:** Literature definitions of spasticity.

Number	Definition
1	‘Disordered sensory‐motor control, resulting from an upper motor neuron lesion, presenting as intermittent or sustained involuntary activation of muscles’ [7, p. 72].
2	‘A motor disorder characterised by a velocity‐dependent increase in tonic stretch reflexes (muscle tone) with exaggerated tendon jerks, resulting from hyperexcitability of the stretch reflex’ [8, p. 137].
3	‘Involuntary muscle hyperactivity in the presence of central paresis’ [9] due to a neurological condition (p. 856).
4	‘Involuntary muscle overactivity, which commonly follows damage to the central nervous system (brain and spinal cord)’ [10, p. vii].

Injection of botulinum toxin type A (BoNT‐A) into affected muscles is accepted to be the gold standard of treatment for focal spasticity management in neuro‐rehabilitation services for stroke, MS and head injury [10]. The Royal College of Physicians guidelines advocate that spasticity should be treated when it causes symptoms or problems for the patient's functional abilities or care [10], and injection would usually be done within an OP clinic appointment, on a 3–6 monthly regular basis to sustain benefits [10].

A recent literature review identified six publications that focused on the impact of the pandemic on the shutdown of BT treatment services for spasticity management [12]. Across these studies, most patients (between 72% and 93%) perceived that their spasticity worsened when their usual consultation appointments for BT treatment were interrupted [12]. The duration of appointment interruption varied from 36 to 75 days in the studies due to different lockdown periods and policies in different countries. Overall, this translated into a mean treatment delay of between 23 and 129 days, as there were difficulties in re‐scheduling patients back into clinic slots because of reduced capacity when centres reopened due to the requirement to adhere to new public health measures [12].

One study in this review evaluated a 6‐week shutdown of a BT OP spasticity clinic service in Germany on a group of 45 patients who had previously been receiving BT injections for an average of 8 years [13]. On average, the shutdown delayed BT injection by 6 weeks. Consequently, 93% of patients reported increased muscle cramps, and 82% reported increased pain with a perceived reduction in quality of life by 40% [13]. In terms of attitude, 66% of participants perceived BT injection as more important than before the pandemic lockdown and all participants rated having access to BT treatment in the long term as either very important or important [13]. This study was based on the utilisation of a basic structured questionnaire focused on six broad domains created by the two authors and administered via an in‐person interview. There is no detail on how the questionnaire was designed and created, indicating a lack of a scientific systematic approach to design, which suggests an overly simplistic approach towards the gathering of subjective data. Hence, result interpretation should be cautionary, and understood within the context of being a rudimentary analysis rather than a high‐quality evidence‐based study.

Subsequent to this, a study evaluated an 8‐week lockdown closure of an OP spasticity treatment centre in Austria using the same questionnaire [14]. A group of 32 patients with a range of diagnoses including stroke and MS who had all been treated with BT injections for spasticity before the pandemic for a median time period of 4 years, with the majority (91%) having an average time period of 12 weeks between injections, took part. On average, the BT injections were delayed by 10 weeks and 97% of participants reported that their symptoms worsened with 80% reporting that this had an adverse impact on their functional abilities [14]. More specifically, 95% of participants reported increased muscle cramps and 60% of participants with spasticity reported increased pain [14]. Overall, participants reported a perceived reduction in quality of life of 62% and in terms of attitude, 78% of participants rated this treatment as more important than pre‐pandemic with 75% rating that continued access to this treatment in the long term was very important [14].

The limited body of research knowledge that currently exists related to how people living with LTNC experienced the pandemic and what if anything could potentially be improved in the future should another global pandemic arise acted as drivers for this study, which aimed to explore how the COVID‐19 pandemic impacted on individuals living with LTNC, their carers and also include the perspectives of a clinical spasticity service lead (CSSL).

In this study, we sought to gain insight into the following research questions:

1. How do people living with an LTNC experience spasticity?
2. How did the COVID‐19 pandemic impact their OP spasticity treatment?
3. What kind of impact did the pandemic have on primary caregivers supporting an individual who usually accessed BT treatment injection via OP clinics?
4. What kinds of challenges, opportunities and barriers were experienced by CSSLs during the pandemic?
5. What might be considered valuable lessons learnt that could potentially enable clinical spasticity service system improvement, especially in the context of a potential future pandemic?

To answer these questions, a qualitative research approach was taken.

## 2 Materials and Methods

### 2.1 Methodology

To gain insight and understanding of what it is like to live with spasticity related to an LTNC with the focus of enquiry being the diverse lived health experiences of patients and carers during the pandemic as well as the challenges experienced by a CSSL, qualitative research methods were chosen as the most relevant and appropriate research approach to take. Qualitative research methods provide a valid means of examining experiences as phenomena [15] and have been used by many health researchers seeking to understand the long‐term nature and impact of health symptoms experienced by people living with long‐term health conditions, for example, ‘long Covid’ [16] and also people living with chronic health diseases such as inflammatory rheumatoid arthritis [17, 18].

### 2.2 Theoretical Framework

There are two theoretical perspectives that underpin this research. The first is described as an interpretivist paradigm [19] and also referred to as a phenomenological perspective [20]. The second theoretical perspective is constructivism [21], where it is understood that there are multiple realities, experienced by individuals, that are all equally valid and can be explored. This paradigm also asserts the theory that knowledge and meaning are generated through the interaction of humans with each other, individual experiences and ideas. In addition, this study has been informed by the literature on the impact of the pandemic on the management of spasticity related to LTNC [2, 12, 13, 14, 22, 23, 24, 25, 26] and qualitative studies focused on the lived experiences of spasticity [27, 28, 29, 30].

### 2.3 Study Design

A qualitative design was selected with semi‐structured interviews [31] conducted by one research team member (K.S.) by telephone [32]. In terms of data collection, there is a growing body of literature, which identifies that telephone interviews are a viable and valuable way to collect rich qualitative data [33, 34].

Three separate interview topic guides were used, related to the specific role of the participants (i.e., patients, carers or professional clinical service lead). The study was conducted from March to the end of April 2022.

### 2.4 Sampling and Recruitment

A purposive sample of 11 participants was recruited (see Table 2 for eligibility criteria). Potential participants were provided with a Participant Information Sheet detailing what the study was about and what their involvement would be, as well as a consent form and debrief document. Recruitment started after research, ethical and organisational approvals had been received. Formal approval was received from the Integrated Research Application System (IRAS) [35] for all health and social care research in the UK (IRAS ID reference: 306570). Ethical approval was received from the NHS Health Research Authority [36], the West London and GTAC Research Ethics Committee, UK (reference: 21/PR/1386) in January 2022, and approval was also received from East Kent Hospitals University NHS Foundation Trust (EKHUFT) Research and Innovation Department (reference: 2021/NEURO/01). HRA‐approved research is conducted within explicit ethical conduct principles defined by the UK Policy Framework for Health and Social Care research [37]. The trial was registered in the National Clinical Trial database (registration reference NCT05435404).

**Table 2:** Participant eligibility criteria.

Individual identity of participant	Eligibility criteria
Individual living with spasticity	Age 18 or over Diagnosed with a long‐term neurological condition (LTNC) Attendance at outpatient (OP) spasticity service clinics of the principal investigator (PI) over the period of 18 months before the pandemic (March 2020) A diagnosis of severe spasticity requiring botulinum toxin injection for spasticity management via the PI OP clinic service at EKHUFT Able to understand the research and provide informed consent
Primary carer for individual living with spasticity	Age 18 or over An individual who accompanied an individual patient (diagnosed with an LTNC), who attended a spasticity service OP clinic with the PI at EKHUFT and who provided care to that patient in a primary care role Able to understand the research and provide informed consent
Clinical spasticity service lead	Consultant physician injector of botulinum toxin for muscle spasticity management (experienced with a minimum of 10 years in this role) Clinical spasticity service lead for patients referred for treatment through the outpatient clinic service at EKHUFT

### 2.5 Consent

Informed consent was obtained from all participants through the completion of consent forms. Participants were assured that all data would be anonymised, stored and handled securely and anonymously in alignment with EKHUFT policy.

### 2.6 Interviews

All interviews were conducted by K.S. (a trained qualitative researcher) who had no previous contact with the patient and carer participants. The interview topic guides were designed following standard practice in qualitative research [20, 21]. This involved combining a comprehensive review of the relevant literature [2, 12, 13, 14, 22, 23, 24, 25, 26, 27, 28, 29, 30] with research team discussion to incorporate relevant professional clinical knowledge, and feedback from professional peer colleagues was also used to refine the guides. The guides used a combination of descriptive and probing questions [31] and incorporated issues that had been identified within early research papers [2, 13, 22]. The majority of the questions were similar for patient and carer groups; however, some additional questions for the patients aimed to explore their perceptions of any impacts on specific aspects of health and well‐being, anxieties in appointments during the pandemic and also regarding perceptions of subsequent access to appointments and any perceived barriers to treatment. The physician topic guide questions were different because the role of a CSSL physician is completely different to that of a patient and carer; however, the questions remained focused on the identified research objectives for this study (Table 3). Interviews were audio recorded, and the duration ranged from 20 to 40 min.

**Table 3:** Interview topic guides for the patient, carer and physician participants.

	Patient	Carer	Physician
	Part 1: Demographic information Gender Age County of residence Ethnicity Diagnosis Date of diagnosis	Part 1: Demographic information Gender Age County of residence What is your relationship to the individual living with spasticity whom you provide care support to? How many years have you been providing care support? What do you provide care support with? Frequency? And an approximate amount of time each day? Could you identify the diagnosis of the medical condition which causes spasticity to be an issue for the person whom you support to? And the approximate year date of diagnosis of the condition causing the issue? What is the age of the person whom you provide care support to? Could you identify the immediate family relationships of the person whom you provide care support to? What are the living arrangements of the person whom you provide care support to? For example, does the person live alone or with others? If with others, who?	Part 1: Demographic information Gender Age Clinical qualification Approximate number of years injecting botulinum toxin for spasticity management
	Part 2: Questions	Key: Shaded boxes indicate identical questions	
1.	Would you say you have problems with muscle spasticity and if so, what kind of problems do you have?	Does the person whom you provide care support to have problems with muscle spasticity and if so, what kind of problems?	How did the COVID‐19 pandemic impact your role in providing patients who needed injection of botulinum toxin for spasticity management treatment?
2.	Does it prevent you from doing anything specific?	Does it prevent the person from doing anything specific?	What kind of challenges did you experience? How did you address these?
3.	Does it cause pain?	Does it cause pain?	What kinds of barriers did you experience? From your perspective, what kinds of barriers did the patients experience?
4.	What would your routine treatment have been before the COVID‐19 pandemic?	How did the person whom you provide care support to manage their spasticity before the COVID pandemic?	What was your perception of health and safety issues and how these could be mitigated during the pandemic?
5.	What happened to your treatment appointments when the pandemic arrived?	How did this change when the pandemic arrived?	What was the transition like from face‐to‐face appointments to telephone and video‐based appointments? Was there enough support and training for staff? Were these appointments effective? Or if not, why not?
6.	If you were unable to access the usual treatment during the pandemic and lockdowns, do you think that this affected your physical health in any way?	How did the pandemic impact the health of the person whom you provide care support for? And did this have any impact on your health?	Would you be able to make any suggestions or give any ideas about what might potentially help to improve both the patient and physician experience of such a clinic outpatient appointment service?
7.	If you were unable to access the usual treatment during the pandemic and lockdowns, do you think that this affected your mobility in any way?	What kind of challenges in relation to spasticity management did the person whom you support experience during the pandemic and lockdowns?	What kind of learning and opportunities do you think might have come through the pandemic with regard to spasticity treatment management?
8.	If you were unable to access the usual treatment during the pandemic and lockdowns, do you think that this affected your mental health in any way?		Do you have any suggestions or ideas on how spasticity could potentially be measured and/or treated during a future pandemic scenario?
9.	If you were unable to access the usual treatment during the pandemic and lockdowns, do you think that this affected your sense of well‐being in any way?		From your perspective, do you think that there is a role for funding research into this area? And would you see potential voluntary assistance from non‐medical and non‐healthcare‐trained individuals as an acceptable potential solution to assist in spasticity management in a future pandemic scenario?
10.	What were your perceptions of potential barriers to accessing the usual botulinum toxin injections for spasticity management during the pandemic?	What were your perceptions of potential barriers to accessing the usual botulinum toxin injections for spasticity management during the pandemic?	Are there any other comments that you would like to add?
11.	Were there any specific worries or anxieties that might have concerned you relating to clinic appointments at this time?	Were there any specific worries or anxieties that concerned you relating to clinic appointments for the person whom you provide care support to at that time?	
12.	Did you access and participate in any telephone outpatient clinic appointments? If so, were these useful?	Did the person whom you provide care support to participate in any telephone and/or video‐based outpatient clinic appointments? If so, were these useful or not?	
13.	Did you access and participate in any video‐based outpatient clinic appointments? If so, were these useful?		
14.	Would there have been an approximate estimate of the length of time that you might not have had the usual outpatient access to treatment for spasticity?		
15.	Did you experience any adverse impacts that you perceive would have been potentially attributable to not having this treatment during the pandemic and lockdowns?		
16.	Because the last lockdown was lifted, have you been able to access clinic outpatient appointments again?		
17.	What has this experience been like for you?	What was the experience like for you of supporting an individual with spasticity issues during the pandemic and lockdowns?	
18.	Do you have any concerns or anxieties regarding these appointments?		
19.	Would you be able to make any suggestions or give any ideas about what might potentially help to improve the patient experience of such a clinic outpatient appointment service?	Would you be able to make any suggestions or give any ideas about what might potentially help to improve the patient experience of such a clinic outpatient appointment service in a future pandemic scenario?	
20.	Do you think that it would be useful for research to be funded into the potential development and testing of a spasticity assessment tool, which could potentially be utilised via a video‐based consultation appointment? Do you think that the potential assistance from non‐medical and non‐healthcare‐trained individuals would be an acceptable potential solution, which could potentially enable the development of a video‐based spasticity measurement system?	Do you think that it would be useful for research to be considered for funding the potential development and testing of a spasticity assessment tool, that could potentially be utilised via a video‐based consultation appointment? Do you think that the potential assistance from non‐medical and non‐healthcare‐trained individuals would be an acceptable potential solution to enable the development of a video‐based spasticity measurement system for a future pandemic scenario?	
21.	What is your perception now of your treatment as compared to previously?		
22.	Can you identify any opportunities for potential improvement of the clinic service?	Could you identify any opportunities for potential improvement of the outpatient clinic service?	
23.	Can you identify any current barriers to treatment? If so, what might help to overcome these?		
24.	Are there any other comments that you would like to add?	Are there any other comments that you would like to add?	

*Note:* Shading indicates which questions are identical.

### 2.7 Project Team

K.S. is a clinical neuro‐physiotherapist and trained qualitative researcher. Although K.S. has awareness and knowledge of the clinical symptoms of spasticity, she is not an injector of BT and has never worked within a clinical spasticity management OP service. D.W. is a senior experienced academic researcher in psychology. R.F. is a consultant neuropsychiatrist with research experience. M.S. is a consultant in Rehabilitation Medicine experienced in clinical and qualitative research. J.K. assisted with manuscript administrative tasks.

### 2.8 Data Management and Analysis

Interviews were transcribed verbatim, and the transcripts were anonymised using pseudonyms. The initial familiarisation phase involved reading over the transcripts, and one single transcript was then coded separately by K.S., D.W., R.F. and M.S. The team then met to discuss coding categories and fidelity to the transcript. After confirming coding fidelity, K.S. went on to code all the transcripts and organised the data into broad categories informed by the theoretical framework. Then, the One Sheet of Paper (OSOP) method [38] of thematic analysis was used to identify all the issues within the text coded in the initial coding framework. This method enables a summary of all issues within a code to be identified and listed on the OSOP and is commonly used within qualitative studies focused on health experiences [39, 40]. At this stage, the project team met to discuss the OSOP. This discussion and reflection enabled progression to the next stage of data analysis, where issues are grouped into broader themes (axial coding) and interpretation of the data can be made [38]. These broader themes were discussed within the research team and were further informed by clinical insights and contextualisation within the relevant peer‐reviewed literature [2, 12, 13, 14, 22, 24, 25, 26, 27, 28, 29, 30]. Voluntary feedback on the identified broad themes was invited from two patient and two carer participants. Following this feedback, the research team met to discuss refinement and finalisation of the themes.

## 3 Results

Demographic details of participants are presented in Table 4. The sample of 11 participants comprised seven people living with LTNC, three people who were primary caregivers and one who was a CSSL. Five participants with LTNC were male and two were female. The majority of participants (four), were living with spasticity related to chronic stroke; two were living with spasticity after spinal cord injury and one was living with spasticity caused by MS.

**Table 4:** Demographic details of participants.

Participant^a^	Age	Gender	LTNC diagnosis and year of diagnosis
Individual living with long‐term neurological condition (LTNC)			
Jake	74	Male	Stroke (2020)
Sally	53	Female	Spinal cord injury (Paraplegia)
Mark	61	Male	Stroke (2019)
Mike	36	Male	Stroke (2016)
Rita	44	Female	Multiple sclerosis (MS) (2002)
Billy	77	Male	Spinal cord injury (Tetraplegia) (2017)
Dennis	76	Male	Stroke (2017)
Carer participant	Age	Gender	Number of years providing care support to a person with LTNC
Fiona	60	Female	2.5
Liz	58	Female	5
Yvonne	68	Female	15
Consultant physician spasticity service lead participant	Age	Gender	Number of years providing spasticity treatment via botulinum toxin injection
Colin	57	Male	24

a All participants were allocated pseudonyms for anonymity.

### 3.1 Thematic Analysis

Six major themes were identified: experience of living with spasticity during the pandemic; impact of the pandemic on patient, carer and clinician health; access to and experience of OP clinic appointments; coping strategies during the pandemic; system improvements; and learning from the pandemic period.

Theme 1 Experience of living with spasticity during the pandemic.

### 3.2 Participants Diagnosed With LTNC

**Figure 1:**
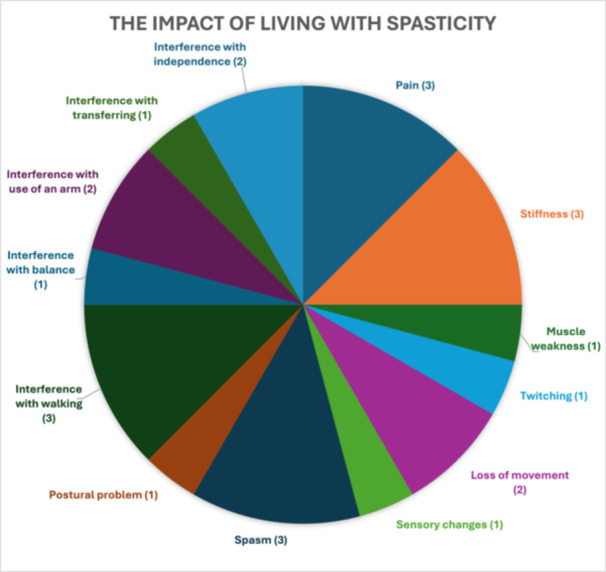
Theme 1: Experiences of living with spasticity during the pandemic—participants diagnosed with long‐term neurological conditions (Key: Number of participant quotes).

Participants identified the main symptoms that they experienced related to living with spasticity as: pain, stiffness, muscle weakness, spasm, twitching, loss of movement, sensory changes and postural problems (Figure 1 and Table 5). Some commented that BT injection usually helped by relaxing muscles, albeit for a temporary period:

I do know it [the BT injection] relaxed my left arm, but when it [spasticity] came back again I was exactly the same.

(Mark)

**Table 5:** Theme 1: Individual experiences of living with spasticity caused by a long‐term neurological condition.

Experience	Comments
Pain	*I get pains in the shoulder and down the upper arm and then sometimes it shoots right down to the hand* (Jake). *I get a lot of nerve pain for which I take Gabapentin, I take the maximum dose* (Rita). *walking is painful* (Rita).
Stiffness	*It [left arm] goes like an iron bar. It just is really as bad as that. And it won't move* (Jake). *Stiffness in my right leg, from my knee downwards to my toes* (Sally). *I get a lot of stiffness in my back as well* (Rita).
Muscle weakness	*Well … through lack of use, you get a lot of waste [muscle]* (Billy).
Twitching	*It's not stiffness, it's twitching, I had a stroke* (Mike).
Loss of movement	*I just don't have any feeling or any control in it at all [left arm]* (Jake). *I cannot move my left arm* (Mark).
Sensory changes	*I cannot feel my left arm. It's dead to me, … numb* (Mark).
Spasm	*I'm aware that my muscles are constantly contorting, like my foot's claw‐like … I know it's spasming all the time* (Sally). *I can't stop it from shaking or spasms* (Mike). *My feet are terrible, the doctor uses Botox at times to release and relax the muscles* (Rita).
Postural problem	*It [left arm] tends consistently to wrap over my midriff and it becomes very, very stiff and solid and I can't move it* (Jake).
Interference with balance	*It [spasticity] affects the balance* (Rita).
Interference with walking	*It [spasticity] does interfere, walking is painful* (Rita).
Interference with use of an arm	*I can't hold anything … in my left arm … I can't hold a drink, or my phone* (Mike).
Interference with independence	*I can't really stand up for too long, and cook, … my aunt will come and help, … chop vegetables and stuff, or my mum, so I rely on other people to … look after me* (Rita). *I have problems with walking and using my right arm. I have assistance with most things* (Dennis).
Interference with transferring	*‘It [spasticity] hinders my transferring*’ (Sally).

However, one participant stated that the experience of having the BT injection was intensely painful and commented that he would not be keen to have any more because of the experience of pain during injection.

It's incredibly painful to achieve but once it [the BT injection] is over … I was relaxed in my left arm for a period of about 3 to 4 months.

(Mark)

### 3.3 Participants Providing Care Support to Individuals Living With an LTNC

**Figure 2:**
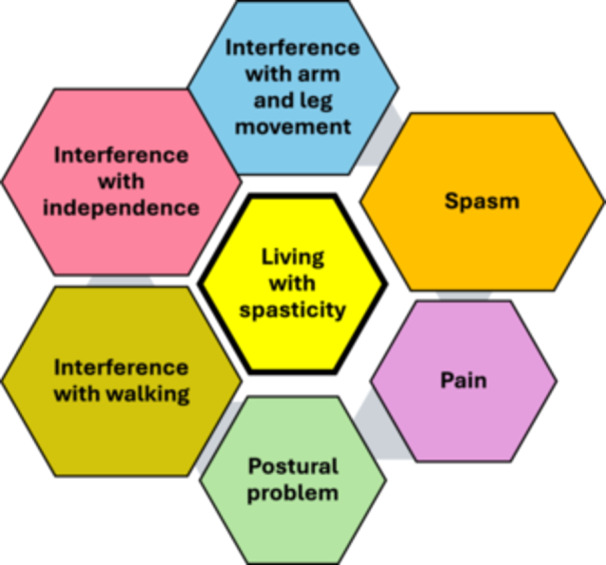
Theme 1: Experiences of living with spasticity during the pandemic—participants providing care support to individuals living with a long‐term neurological condition.

These participants described the main symptoms related to spasticity experienced by the individuals they cared for as pain, spasm and postural problems, which interfered with arm and leg movement, walking and independence for the affected individuals (Figure 2 and Table 6).

**Table 6:** Theme 1: The experiences of carers supporting individuals diagnosed with an LTNC living with spasticity during the pandemic.

Experience	Comments
Impact of spasticity	*It's a total spasticity in his right arm and he needs assistance with all tasks. So that's personal care, toiletries and ensuring, you know, like food and cooking, everything, he's unable to do anything like that for himself. And he needs assistance to stand* (Liz). *The right leg goes into spasm or it shakes and he can't use the right arm at all* (Yvonne). *It [spasticity] makes it very difficult for him to walk properly, feed and do basic hygiene* (Yvonne). *He has pain in the left arm and doesn't have voluntary control…so there's a tendency for his hand to curl up* (Fiona). *He can't use his left hand or arm at all now* (Fiona).

One carer expressed that there had been a worsening of spasticity for the individual for whom she provided care, during the pandemic, when there was no access to the regular BT injection treatment OP appointments, which she commented had effectively compromised the safety for both.

The stiffness increased [right arm and leg] and the shaking increased, which makes his walking … I mean I used to be able to walk him with an aid, with me helping him and a crutch, from one place to another. And it became quite difficult because with a shaking leg it was not always safe.

(Yvonne)

Theme 2 Impact of the pandemic on patient, carer and clinician health.

Participants diagnosed with a LTNC and those supporting individuals living with a LTNC articulated a common experience of negative impacts on their physical and mental health, with associated frustration, anxiety and stress. Some carers experienced extremely high stress related to the loss of outside agency carers, which imposed an increased burden of care on them and caused adverse health impacts:

It [the increased physical efforts related to providing care for an individual with a LTNC during the pandemic] affected my hip – which became painful and I am still seeing a physio now for it’.

(Yvonne)

I was run down, I didn't really have any breaks from it, it was 24/7.

(Liz)

### 3.4 CSSL Perspectives

The participant who had worked on the clinical front line during the pandemic articulated a broader perspective:

It [the pandemic] affected them [patients] profoundly. First of all during lockdown, face to face clinics were stopped, then frequently when the face to face clinics started again, because of the fear by the patient or their relatives, they were reluctant to come to the hospital to get their injection … therefore a lot of patients missed out on BT.

(Colin)

Community therapy teams that provide support for spasticity management also stopped visiting patients during the pandemic period, which had an adverse impact on patient health:

the community teams were diverted to Covid patients in the hospitals or in primary care, so patients with spasticity management needs became much worse much more quickly than they would have otherwise.

(Colin)

After the pandemic period, the accumulated backlog of patients who required BT treatment created an enormous system challenge, which created workforce pressure to provide more clinic appointments with the same finite number of staff. This system pressure caused stress and anxiety for clinical staff responsible for service provision:

How do I go through this large number of patients who have accumulated over these 2 years or so? … doing extra clinics meant that there was less time available to do other work.

(Colin)

Theme 3 Access and experience of OP BT clinic appointments.

Participant experiences and attitudes varied regarding OP appointments and issues encountered. Appointment cancellation due to staff illness was identified as a barrier and the difficulty and delays with re‐scheduling appointments were a source of frustration and concern:

It [appointments] does create panic and distress when I've got to … worry about those things, are we going to make it, are we going … it certainly does cause me anxiety.

(Jake)

Participants had all experienced telephone appointments, and some had also experienced video appointments during the pandemic period. After clinics reopened and face‐to‐face appointments started again, perspectives were mixed. Some participants stated that the space and wheelchair access were fine:

It was easy, it felt very safe going into the unit.

(Sally)

Whereas another commented:

It was a very small room and I was worried that I was going to get it [Covid].

(Rita)

### 3.5 CSSL Perspectives

Following the lockdown period, the perception of the risk of potentially catching COVID‐19 meant that sometimes patients and carers were reluctant to attend an OP appointment:

the fear of patients that if they come to the hospital then they will get infected … and on occasion they're [carers] reluctant to bring the patient to the hospital as well, so there is justifiable fear because there was no guarantee that they will not get infected if they come to the hospital.

(Colin)

Resources were identified as critically important for improving the experience of an OP appointment:

More additional resources need to be allocated, not just to the doctor but also to the administrative staff, the ambulance and clinic nurse.

(Colin)

Theme 4 Coping strategies during the pandemic.

Individual participants living with a LTNC and carer participants coped in different ways, with some more able to adapt than others:

We managed to do all the exercises ourselves at home …to maintain abilities.

(Dennis)

It hasn't stiffened up [arm] because we carried on with the regular stretching exercises that we've been given … to maintain his movement and abilities.

(Liz)

Taking a pragmatic stoic attitude was adopted by one participant who reflected:

I mean it was difficult, because obviously we had reduced access to hospitals and things like that, but you know, I just carried on and got on with it.

(Rita)

### 3.6 CSSL Perspectives

The use of video appointments for spasticity assessment was tried but was acknowledged to be sub‐optimal:

Eventually we did manage to assess patients through video, which cannot be optimal, but we were stuck with such a system but we can't inject through video.

(Colin)

Theme 5 System improvements.

Participants made a few suggestions on possible improvements, for example, sending a reminder message or letter nearer to the actual appointment date:

I think that giving me a note, a letter, four months before the actual date is not extremely helpful, considering the fact that I've only half a brain working … so we don't receive any reminders nearer the date … so it would be really helpful if there was a reminder.

(Jake)

It was suggested to inform the patient in advance of the appointment whether a BT injection would be given and to co‐ordinate some form of direct communication with the relevant physiotherapy team:

what they don't seem to tell me is whether it is for an injection or whether it's just a review … then at least I can talk to the physios and tell them the exact date and make an arrangement with them to come in that window, to maximise the effectiveness of the of the BT.

(Jake)

### 3.7 CSSL Perspectives

There was a lack of national and international guidelines regarding specific health and safety risk assessment for BT injection during the pandemic period, which created unprecedented challenge for clinicians who provided this service:

my perception was we try and develop evidence of how risky it is … but that requires research and funding and we couldn't get the funding, therefore we couldn't do that.

(Colin)

Service development via innovation with the utilisation of video technologies was identified as a possible mechanism for system improvement:

video ways assessment, … there needs to be more research to actually develop that to make it more acceptable as well as more scientifically sound.

(Colin)

There would be associated training needs around this for staff, patients and carers, which would need to be addressed:

not all patients will have the technical know how, so there needs to be targeted support for that as well.

(Colin)

Research into the assessment and measurement of spasticity via video was identified as an opportunity to potentially improve service options in the future:

we need to develop a research protocol for that measurement and that assessment, through video.

(Colin)

Theme 6 Learning from the pandemic period.

### 3.8 CSSL Perspectives

The CSSL participant reflected that the management of spasticity for people diagnosed with LTNC had been severely affected during the pandemic and that he had observed a range of adverse health impacts for such patients and their carers.

it has confirmed one point, which is continuous and regular management of spasticity is necessary to reduce complications … like pain, deformities, contractures and reduced mobility. That, we have now seen it happen.

(Colin)

In addition, it was apparent that the stopping of a BT OP clinic service for any period of time would automatically result in a waiting list backlog and how could this backlog waiting list be better managed.

it was a big problem, so we need to have service planning, how do you handle it? For example, we need to develop a sort of, a traffic light system that when there is a backlog, how do you prioritise urgent patients for urgent injection? So those things have to be researched and developed for service planning.

(Colin)

Further learning was that communication was affected:

communication does get affected when you are wearing PPE [personal protective equipment] and you just have to think innovatively how will you get around it?

(Colin)

The importance of carer support also became evident:

the carer's training does matter, it does affect the … clinical care of the patient.

(Colin)

## 4 Discussion

### 4.1 Summary of Key Findings

This qualitative study has provided insights into how a group of individuals living with a diverse range of LTNCs and carers supporting such individuals experienced living through the COVID‐19 pandemic. Spasticity as a phenomenon experienced by participants in this study had an adverse impact on health and a range of everyday tasks and activities, which was associated with reduced independence. During the pandemic, carer participants experienced a higher level of care burden, and this impacted on their physical and mental health. In addition, important insights have been gained from a CSSL, who observed a rapid deterioration in the abilities of patients with LTNC living in the community to cope, related to community resources being re‐deployed within the acute hospital setting. The CSSL also experienced adverse stress and health impacts related to the increased system pressure for more OP clinics to deal with the waiting list backlog created by the pandemic. Coping strategies varied and participants described a variety of ways that system improvements could potentially be addressed in the future. A key insight was how the loss of community resources created immensely difficult challenges for patients and carers and also regarding service provision and sustenance after the pandemic. The experiences of CSSLs during the pandemic are important, and the lack of research funding for these clinicians to undertake relevant and much‐needed research directly related to clinical practice reflects a flawed funding research system. Broader learning from reflection centred on the importance of and greater challenges to communication experienced; the importance of OP clinical services to manage spasticity; the need for funded research for clinicians working in this area; the need for service planning and also greater recognition for the role of the carer.

### 4.2 Comparison With Other Relevant Research Literature

People living with LTNC are understood to be a diverse group, and many who are living with MS [41] and chronic stroke [42] experience spasticity, which negatively impacts their everyday abilities. This aligns with accounts given by participants in our study, and it is salient that in a recent large study of 262 participants living with MS, 23% (59) identified that spasticity was the most frequently debilitating symptom experienced in their lives [41]. After spasticity, among the other limiting symptoms reported were muscle weakness (20% [53]), balance (15% [40]) and pain (7% [20]) [41]. This validates and aligns with participant narratives within our study, where the main symptoms related to spasticity, described as problematic, included pain, muscle weakness and balance issues. In addition, participant narratives in our study identified further negative experiences related to spasticity: stiffness, spasm, twitching, loss of movement, sensory changes, postural problems, interference with walking, arm and leg movement and independence (Figure 1).

A study of 30 patients living with post‐stroke spasticity, who receive BT injection treatment identified their most common debilitating symptoms as spasms, sleeping issues, stiffness and pain [42], three of which align with our findings and validate this study. It is also salient to note that within another qualitative study of 14 people living with chronic disabling spasticity after stroke (which required BT treatment), a comparable theme was identified as ‘spasticity‐related impairments and activity limitations’ [42, p. 3688]. This further supports and validates the findings of Theme 1.

The experience of living with spasticity, impact and coping strategies were explored in a recent study of 13 people living with MS [5], and our findings align with two of the themes identified within this study: living with spasticity and coping with spasticity, which endorses the thematic insights from this research.

Further insight into the struggles of living with spasticity is described within a qualitative study of 14 people living with hereditary spastic paraplegia [27], which refers to how difficult balance and walking can become. This research resonates with participant narratives within our study, where participants and carers expressed how balance and walking were negatively affected by spasticity.

Spasticity that causes a postural change in the position of an arm and how that negatively impacts functional independence has been explored in a recent study [43]. This study concluded that the postural pattern of an arm affected by spasticity, especially one that is folded and stuck across the body (Pattern 1, p. 552) [43], is related to and impacts the patient's independence. This endorses the reported experiences by Jake, Mike and Dennis, who all experienced a reduced ability to use an arm affected by spasticity post stroke.

The deterioration in the abilities of individuals living with LTNC in the community during the pandemic observed by the CSSL and Yvonne (carer) fit in with two research studies, which identified pain and muscle cramps as being more problematic after the shutdown of BT spasticity clinic treatment services [13, 14].

### 4.3 Comparison With Other Relevant COVID‐19 Pandemic and Chronic Disease Literature

The COVID‐19 pandemic had an enormously negative impact on peoples’ health in the UK, and many people (including NHS staff) who were exposed to the virus (pre‐vaccine manufacture and availability) died [44]. Resources including NHS staff were severely depleted and stress, anxiety and burnout were reported across medical and healthcare staff groups [44]. Our study adds important insights relating to the complexity of challenge for a CSSL, who was responsible for specialist OP service delivery when system priorities changed nationally to acute in‐patient care due to the pandemic.

Within the United States, where stroke survivors are understood to be living with a chronic disease, the impact of the COVID‐19 pandemic has been acknowledged to be extremely profound with direct and indirect health effects on people living with chronic disease, and questions have been raised about how these vulnerable individuals can safely access health care [45].

In Italy, post‐pandemic, some clinical recommendations have been made regarding how OP services for patients who usually receive BT for spasticity treatment could potentially be reorganised and improved [22]. Measures suggested include re‐organisation of waiting rooms, adequate spacing of patients and the use of correct Personal Protective Equipment. Measures to screen and assess for priority of treatment need have also been suggested by using telephone and video tools. Our findings challenge the effectiveness of video assessment and identify that research would need to be done to explore how such a process could potentially work. Funding research for CSSLs to explore and develop UK guidelines in this area is fundamentally needed.

### 4.4 Reflective Learning

Reflective learning from this study within the NHS and wider pandemic context is that effective management of spasticity is important to support and enable the independence of individuals affected by LTNC. If the abilities of an individual living with a LTNC decline due to a lack of access to appropriate and timely BT injection treatment (as was the case during the pandemic), then this may also have an associated adverse impact on the health of any primary carer providing care to the person. If a primary carer's health decreases then they will become less able to continue in that role. The ultimate outcome in this scenario could be that the individual with a LTNC may need support via an external care agency or even admission to a residential care home. In this example, a need to access social care funding could arise, which could have much higher financial costs than prioritising funding for research into clinical spasticity service planning and improvement. This is one reason that funding of research into clinical spasticity services needs to be prioritised and the importance better understood as well as appreciating that the ramifications of not doing this, could have much higher financial costs in the long term. To date, there have been numerous variants of the SARS‐CoV‐2 virus identified that continue to infect individuals when exposed, whereas there seems to be little impetus now within the United Kingdom to explore and consider the development of clinical evidence‐based guidelines within this specialist area. Our reflection is that service planning in advance needs to be supported and undertaken within the NHS and that this should be underpinned by funded research and systematic clinical evidence review. A scoping review on pandemic preparedness literature reported that among the best practices identified that ‘post pandemic reflections and learning for resilience … can improve future preparedness and evidence‐based policy making’ [46, p. 15]. Our reflections align with this best practice, and we see this research as a contribution towards highlighting the importance of clinical spasticity management for people with LTNC, emphasising the vital role of the CSSL in enabling this goal and identifying the importance of funded research allocated to CSSLs working in this area.

### 4.5 Strengths and Limitations

To our knowledge, this is the only qualitative research study that has included the experiences of a CSSL working within the NHS during the COVID‐19 pandemic and therefore it provides a unique contribution towards research knowledge. The research team included clinicians from different clinical backgrounds as well as an academic professor, which enabled multidisciplinary and interdisciplinary discussion and interpretation of the findings. The heterogeneity of participants diagnosed with LTNC enabled diverse perspectives to be gathered from participants. Perspectives were gained across three different participant groups (patient, carer and CSSL), which enabled a broader understanding of the themes identified. However, the study has some limitations. The first relates to the reliance on participant memories when the interviews were conducted, in which both patient and carer participants identified that they found it difficult to remember the detail of their experiences at that time. The length of time it took to gain all the relevant organisational and ethical approvals was also a factor in this respect. The second relates to the lack of funding for this research. This meant that patient and public participation could only be achieved through voluntary invitation and that recruitment was restricted to a single clinical spasticity OP service. Had the research been funded, then we would have been able to seek and recruit potential participants across all three groups outside of this OP service.

## 5 Conclusion

This study has provided unique insights into the experiences of individuals living with spasticity who required BT injection during the pandemic and their primary carers. The experiences of a CSSL have also been identified, and a post‐pandemic reflection on learning has been included as best practice.

## Author Contributions

**Mohamed Sakel:** conceptualisation, investigation, writing–review and editing, formal analysis, data curation. **Karen Saunders:** investigation, writing–original draft, methodology, validation, visualisation, writing–review and editing, formal analysis, project administration, data curation. **Rafey Faruqui:** writing–review and editing, formal analysis. **Jamie Keene:** project administration. **David Wilkinson:** formal analysis.

## Ethics Statement

Formal approval for this research was received through the IRAS system (reference: 306570). Ethical approval was received from the NHS Health Research Authority (HRA) West London and GTAC Research Ethics Committee (REC reference 21/PR/1386). HRA approved research is conducted within explicit ethical principles defined by the UK Policy Framework for Health and Social Care Research, which includes reference to the Declaration of Helsinki. Institutional approval was also received from East Kent Hospital University NHS Foundation Trust (EKHUFT) with Research and Innovation (reference: 2021/NEURO/01). All participants provided informed written consent via completion of consent forms in alignment with EKHUFT policy. This trial has been registered on the National Clinical Trial database (registration number: NCT05435404.

## Conflicts of Interest

The authors declare no conflicts of interest.

## Data Availability

The data that support the findings of this study are available on request from the corresponding author. The data are not publicly available due to privacy or ethical restrictions.
